# Semi-Supervised Medical Image Segmentation Guided by Bi-Directional Constrained Dual-Task Consistency

**DOI:** 10.3390/bioengineering10020225

**Published:** 2023-02-07

**Authors:** Ming-Zhang Pan, Xiao-Lan Liao, Zhen Li, Ya-Wen Deng, Yuan Chen, Gui-Bin Bian

**Affiliations:** 1 School of Mechanical Engineering, Guangxi University, Nanning 530004, China; 2 School of Electronic and Information Engineering, Tongji University, Shanghai 200092, China; 3 Institute of Automation, Chinese Academy of Sciences, Beijing 100190, China

**Keywords:** medical image processing, pelvic CT segmentation, semi-supervised learning, interpolation consistency regularization, dual-task consistency

## Abstract

Background: Medical image processing tasks represented by multi-object segmentation are of great significance for surgical planning, robot-assisted surgery, and surgical safety. However, the exceptionally low contrast among tissues and limited available annotated data makes developing an automatic segmentation algorithm for pelvic CT challenging. Methods: A bi-direction constrained dual-task consistency model named PICT is proposed to improve segmentation quality by leveraging free unlabeled data. First, to learn more unmarked data features, it encourages the model prediction of the interpolated image to be consistent with the interpolation of the model prediction at the pixel, model, and data levels. Moreover, to constrain the error prediction of interpolation interference, PICT designs an auxiliary pseudo-supervision task that focuses on the underlying information of non-interpolation data. Finally, an effective loss algorithm for both consistency tasks is designed to ensure the complementary manner and produce more reliable predictions. Results: Quantitative experiments show that the proposed PICT achieves 87.18%, 96.42%, and 79.41% mean DSC score on ACDC, CTPelvic1k, and the individual Multi-tissue Pelvis dataset with gains of around 0.8%, 0.5%, and 1% compared to the state-of-the-art semi-supervised method. Compared to the baseline supervised method, the PICT brings over 3–9% improvements. Conclusions: The developed PICT model can effectively leverage unlabeled data to improve segmentation quality of low contrast medical images. The segmentation result could improve the precision of surgical path planning and provide input for robot-assisted surgery.

## 1. Introduction

Preoperative pelvic Computed Tomography (CT) segmentation is a key technology in computer-assisted surgery and minimally invasive surgical robot navigation [1,2,3,4,5]. The semantic segmentation results of soft and hard tissues can provide supplementary information on pathology and anatomy, help accurate diagnosis, and provide surgical image guidance [3]. Furthermore, the segmentation results can support the surgical path planning and postoperative evaluation [4,5].

**Figure 1:**
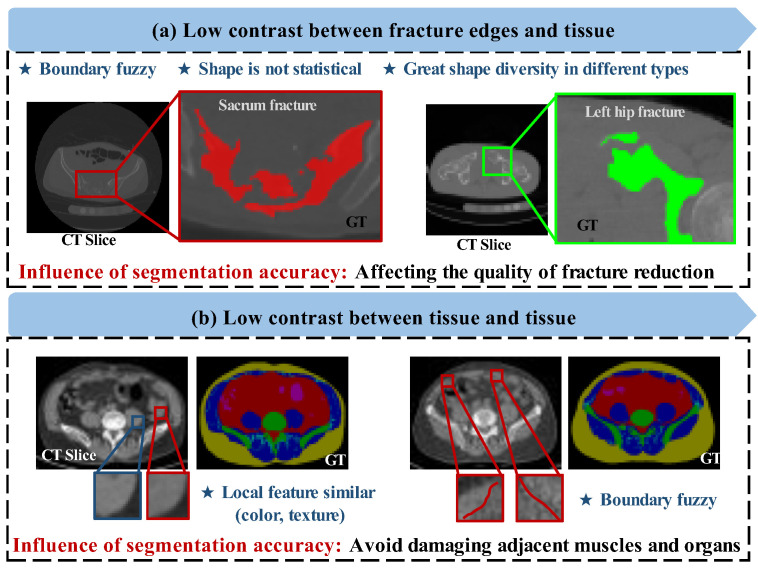
Some difficult samples of pelvic CT slices. (**a**) Low contrast between fracture edges and tissue. (**b**) Low contrast and local similarity between tissue and tissue. The boxes are the “interested region”, and the colors used are their corresponding GT marking color. The difficulties of segmentation are listed with asterisks.

However, developing an automatic segmentation algorithm for soft and hard tissue of pelvic CT faces many challenges. The major challenge is the similarity of gray-scale features and local texture between tissues. As shown in Figure 1a, the contrast between soft and hard tissues is relatively low. The CT intensity of the fracture edge is similar to that of soft tissue, and the fracture edge is fuzzy. In addition, the shape of fracture blocks cannot be predicted, which makes it hard to segment according to prior knowledge. As shown in Figure 1b, the contrast between different soft tissues is relatively low. The local features such as color (threshold intensity) and texture are highly similar for muscles and other pelvic cavity organs. In computer-assisted pelvic surgery, inaccurate recognition and segmentation of soft tissues may damage organs, resulting in a severe iatrogenic injury.

In the past years, the convolutional neural network (CNN), with its strong non-linear modeling capability [6] and capturing non-explicit feature capability [7], has demonstrated remarkable results in medical image segmentation [8]. However, training a well-trained pelvic CT segmentation model is usually at the expense of requiring a large-scale high quality-per-pixel annotated dataset [9,10]. Unlike natural images, obtaining a large, labeled dataset in the medical field is extremely difficult. The number of samples in the dataset limits the complexity of the networks [11]. Stacking complexity for the network, such as network depth and additional encoder structure, may become prone to a redundant use of information and give rise to over-fitting performance on the source dataset [12,13]. Therefore, increasing the complexity of CNN may not be the most effective choice for pelvic anatomical segmentation with a small dataset or even without a dataset.

An alternative solution to reduce the labeled data burden is the semi-supervised learning (SSL) method [14]. In recent studies, interpolation consistency training (ICT) outperforms other state-of-the-art methods in both natural images [15] and medical images [16], making it an appealing approach to SSL. The ICT method augments the input samples in a pixel-level interpolation perturbation manner. It increases the ability to capture detailed features by resisting the subtle differences [17,18] between the interpolation augmentation data. However, this manner ignores the helpful underlying information inside the data itself. Furthermore, the consistency regularization method usually has loss caused by some unpredictable perturbations [19]. The perturbation may strengthen the noise interference to the network learning, resulting in the wrong classification of pixels. Some studies [20,21] showed that integrating network prediction in different training processes can improve the quality of semi-supervised prediction. Therefore, this work designed a pseudo-label supervision module as an auxiliary supervision task, focusing on the undisturbed feature structure of the original unlabeled data and integrating the prediction consistency of the pseudo-label supervision module and the interpolation module to produce more reliable predictions.

Specifically, the proposed interpolation-based pseudo-supervision (PICT) consists of two consistency tasks: the interpolation consistency task and the pseudo-supervision task. The former task encourages the teacher model predictions of pairwise unlabeled interpolation data to be consistent with the student model interpolation prediction of unlabeled data, which is a pixel-level and model-level consistency. The latter task utilizes the unlabeled predictions of the teacher model as pseudo-labels to conduct supervision on student unlabeled outputs and further encourages consistency between the two network predictions, which is both the model-level and task-level consistency. The two joint tasks act in a complementary manner and successfully tackle both low-contrast tissues and high-similar feature problems. The contributions of this paper are summarized as follows:

- A bi-directional constrained dual-task consistency method is proposed; the PICT enhances the ability to learn data features by resisting subtle differences at pixel, model, and task level, and can effectively capture and infer the tissue semantic feature in the low contrast area of pelvic CT.
- A pseudo-supervision module was designed as an auxiliary supervision task to learn the underlying information of original unlabeled data without perturbations, so as to constrain some false predictions of interpolation.
- A multi-object pelvic dataset annotated by experts was developed, which contains 100 CT and subdivides the muscles, tissues, and bones with extremely low contrast into seven categories. PICT achieves state-of-the-art performance in three challenging medical datasets: ACDC, CTPelvic1k, and the individual dataset Multi-tissue Pelvic.

## 2. Related Work

The SSL method can be roughly grouped into four categories: adversarial learning method, self-training method, co-training method, and consistency regularization method [22].

### 2.1. Adversarial Learning Method

The adversarial method is a process in which two networks compete against each other [23]. One is the generation network, the other one is discrimination network. The generation network confuses the discrimination network by generating false data. The task of the discriminating network is to distinguish whether the data comes from the generator or the truth. However, most studies focus on small-resolution data and small-scale data. Medical data like pelvic CT images are usually accompanied by low contrast, complex, and large-scale characteristics, making it more challenging to generate fine details for the generator [24].

### 2.2. Self-Training Method

The self-training method usually uses a pre-trained model to generate pseudo-labels of unlabeled data to expand the limited labeled dataset and then train the model until the performance improvement can be ignored [25]. However, in the field of medical imaging, large public datasets are scarce, so it is difficult to match a suitable pre-trained model. Due to the difference in distribution and the mismatch of samples, it is not simple to extend semi-supervised to cross-domain data [26]. The second disadvantage is that the quality of pseudo-labels is generally less reliable in early training. The incorrect prediction may be strengthened, resulting in the worse performance of the model [27].

### 2.3. Co-Training Method

The co-training method usually trains two models with different initializations simultaneously and encourages them to take each other’s predictions as pseudo-supervision signals [28]; ref. [29] put forward the view that training models with strong tags and pseudo-tags may lead to disordered back propagation. Moreover, this method also has the disadvantage of self-training, that is, the low confidence of early pseudo-labels. Not only that, the co-training method usually requires high computational costs and time.

### 2.4. Consistency Regularization Method

The consistency regularization method follows the assumption that the predictions of the same input should be consistent, which is a method of expanding dataset and avoiding overfitting through technical means, such as noise perturbations [30], data augmentation [31], and mixing up [32]. Tarvainen [33] designs a mean teacher model, which considers the exponential moving average (EMA) of student model parameters as the teacher model parameters. However, the random perturbations of the consistent models are inefficient in high-dimensional space, because only a tiny proportion of input perturbations can push the distribution decision boundary of unlabeled data into the low-density region, which may result in the loss of universality [34]. Vikas Verma [15] further introduced a pixel-level consistency and confirmed the effectiveness of this method, namely interpolation consistency training. This method enforces the low-density separation to achieve sample aggregation of the same category and the separation of samples of different categories, giving rise to the accuracy of model recognition.

However, the existing consistency methods basically enforce consistency on the unlabeled perturbations data, and do not make use of the original unlabeled data structure characteristics. The main disadvantage is that some perturbations may not be in the adversarial direction, in which the network is liable to misclassify the pixels, leading to the loss of generalization ability [16,35]. Thus, this work designs a pseudo-supervision module as an auxiliary task, with the original unlabeled data as input of the student model and teacher model, to achieve the consistency of the data underlying information between the two models, which can limit the error prediction of the interpolation data. Next, the method proposed in this paper will be described in detail.

## 3. Materials and Methods

### 3.1. Overview of Network Architecture

**Figure 2:**
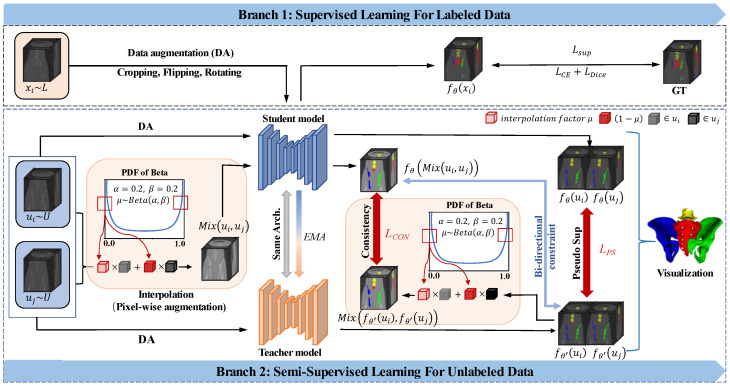
Schematic view of the proposed bi-directional constrained dual-task consistency method on CTPelvic1k dataset. The framework consists of two branches: supervised learning and semi-supervised learning. The below semi-supervised part is the combination of interpolation consistency regularization task and pseudo-label supervision task. The backbone models share the same architecture, and the weight of teacher model is the exponential moving average (EMA) of the student model. The “PDF” in the interpolation part represents the probability density function of beta distribution, “α” and “β” are the parameter of beta distribution, “μ” is the interpolation factor.

The overall framework is illustrated in Figure 2, which consists of two feature extractors, the supervised learning branch and the semi-supervised learning branch. The two feature extractors share the same backbone of U-Net architecture and follow the spirit of mean teacher. The semi-supervised branch contains two mutually constrained tasks: the interpolation consistency regularization task and pseudo-supervision task. Assuming that the training set D consists of N labeled data and M unlabeled data, they are denoted as $D_{L}={\left\{\left(x_{i},y_{i}\right)\right\}}_{i=1}^{N}$ and $D_{U}={\left\{\left(x_{i},y_{i}\right)\right\}}_{i=1}^{N}$, respectively. For the 2D dataset, $x_{i}\in R^{H\times W}$ represents the input pixel, and $y_{i}\in {\left\{0,1\right\}}^{H\times W}$ represents the corresponding ground-truth annotation. The goal of the approved PICT is to minimize the following combined function:

(1) $$\underset{\theta }{\min}\underset{supervised\;loss}{\underset{︸}{\sum_{i=1}^{N}L_{sup}}}+\lambda_{d}\left(t\right)\underset{semi-supervised\;loss}{\underset{︸}{\sum_{i=N+1}^{N+M}(L_{con}+L_{ps})}},$$

where $L_{sup}$ is the supervised loss only used for labeled data $D_{L}$, $L_{con}$ is the consistency loss designed for interpolation data, and $L_{ps}$ is the pseudo-supervision. Consequently, the PICT optimizes the network in a semi-supervised manner by jointly using labeled data, unlabeled data, and interpolation data. Here, we introduce the time-dependent Gaussian warming-up function [33] as a balance factor to control the trade-off between the supervision loss and semi-supervised loss,

(2) $$\lambda_{d}\left(t\right)=exp\left(-5{\left(1-t/t_{max}\right)}^{2}\right),$$

where t denotes the current training step, and $t_{max}$ is the maximum training step.

### 3.2. Supervision Task Design

The supervision part conducted the combination of pixel-wise Dice Loss $L_{Dice}$ and Cross-Entropy (CE) Loss $L_{CE}$ to evaluate the quality between the student network output and ground truth label and minimize the following loss function to update the weight:

(3) $$L_{sup}=\sum_{i=1}^{N}L_{Dice}\left(f\left(x_{i};\theta \right),y_{i}\right)+\sum_{i=1}^{N}L_{CE}\left(f\left(x_{i};\theta \right),y_{i}\right)$$

where the $f\left(x_{i};\theta \right)$ and θ represent the segmentation confidence maps and weights of the student model.

### 3.3. Interpolation Consistency Regularization Task Design

**Figure 3:**
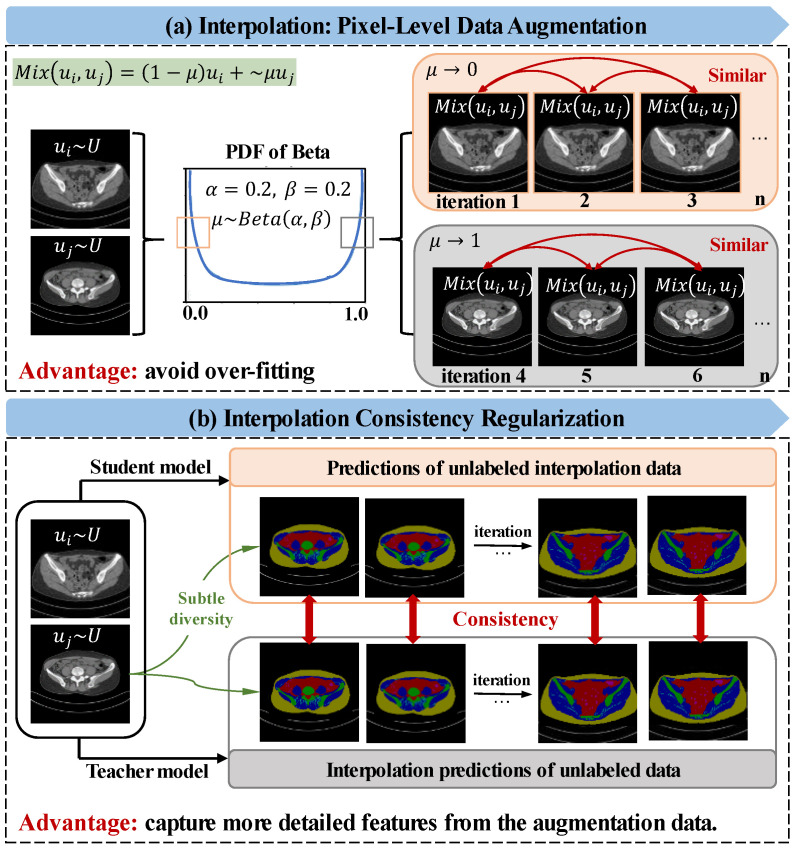
Illustration of interpolation consistency regularization, taking the Multi-tissue Pelvic dataset as an example. (**a**) describes the data augmentation process by pixel level interpolation, “μ” is the interpolation factor and follows the Beta distribution. The “PDF” represents the probability-density function of Beta distribution, and “α” and “β” are the parameters of beta distribution. (**b**) is the interpolation consistency regularization process.

The interpolation task considers two unlabeled image data-point and interpolates the two unlabeled image data-point

(4) $$M_{\alpha }\left(x_{i},x_{j}\right)=\sum_{i,j=N+1}^{N+M}\left[\mu x_{i}+\left(1-\mu \right)x_{j}\right]$$

where $M_{\alpha }\left(x_{i},x_{j}\right)$ represents the unlabeled interpolation data, and μ is the interpolation factor and follows the beta distribution $\mu \sim \mathrm{Beta}\left(\alpha ,\beta \right)$, for α,β∈*(0, ∞)*, μ∈*[0,1]*. α and β are the hyper-parameters of the interpolation factor, which controls the strength of interpolation between data pairs; α is set to be consistent with β [36], following the hyper-parameters setting [16] so that each update randomly generates μ from $\mathrm{Beta}\left(0.2,0.2\right)$. When μ tends to zero, the interpolated image is more similar to data point 1; when μ tends to 1, the interpolated image is more similar to data point 2. This pixel-level interpolation generates the augmented data, which can effectively avoid data overfitting, as shown in Figure 3a. Next, consistency regularization is applied between the unlabeled interpolation predictions of the student model and the interpolation of the teacher model unlabeled predictions, as can be seen in Figure 3b. The network enhances the ability to learn the detailed feature of data by resisting the subtle pixel-level difference of augmented data. In a nutshell, we first interpolate the input of the student model, then interpolate the output of the teacher model, and finally force the output of the two parallel models to be consistent by $L_{2}$ Loss:

(5) $$L_{con}=\sum_{i,j=N+1}^{N+M}{\left\|f\left(M_{\alpha }\left(x_{i},x_{j}\right);\theta \right)-M_{\alpha }\left(f\left(x_{i};\theta^{\prime }\right),f\left(x_{j};\theta^{\prime }\right)\right)\right\|}^{2},$$

where $f\left(\cdot ;\theta^{\prime }\right)$ and $\theta^{\prime }$ represent the segmentation confidence maps and weight of teacher model, respectively. Here, the teacher weights of $\theta^{\prime }$ are updated by an exponential moving average (EMA) of the student weights. $M_{\alpha }\left(f\left(\cdot ;\theta^{\prime }\right),f\left(\cdot ;\theta^{\prime }\right)\right)$ on the right side of the equation represents the interpolation of the teacher model outputs:

(6) $$M_{\alpha }\left(f\left(x_{i};\theta^{\prime }\right),f\left(x_{j};\theta^{\prime }\right)\right)=\sum_{i,j=N+1}^{N+M}\left[\alpha f\left(x_{i};\theta^{\prime }\right)+\left(1-\alpha \right)f\left(x_{j};\theta^{\prime }\right)\right],$$

### 3.4. Pseudo-Label Supervision Task Design

To resist the feature interference caused by some interpolation perturbations, an auxiliary task of pseudo-label supervision was introduced to focus on the feature structure of the original data without perturbations:

(7) $$L_{ps}=\sum_{i=N+1}^{N+M}L_{Dice}\left[f\left(x_{i};\theta \right),P\left(f\left(x_{i};\theta^{\prime }\right)\right)\right],$$

where $P\left(f\left(x_{i};\theta^{\prime }\right)\right)$ is the max label map of teacher model segmentation confidence map, that is, the pseudo-segmentation map. The pseudo-segmentation map of the teacher model will act as the pseudo-label for guiding the student model. The approach uses the idea of transforming the prediction diversity as auxiliary supervision signals to strengthen the training of the two parallel networks.

### 3.5. Data and Label Preprocessing

This section introduces information about the test datasets: Multi-tissue Pelvic dataset, CTPelvic1k dataset [37], and Automated Cardiac Diagnosis Challenge (ACDC) dataset [38]. More detailed statistics of these three datasets are listed in Table 1.

#### 3.5.1. Multi-Tissue Pelvic Dataset

To evaluate the anatomical segmentation performance of proposed PITC methods for pelvic CT, 100 slices with the size of 512 × 512 pixels from the total of 6251 slices were randomly selected to form the Multi-tissue Pelvic dataset. The window width of all slices was set to 60 HU, the window level was set to 500 HU, and then a median filtering algorithm was used to reduce noise. Under the medical annotation software Pair, seven categories in each slice were manually delineated by three experienced radiologists. The seven categories were background, miscellaneous intra-pelvic content (MIPC), bone, muscle, subcutaneous adipose tissue (SAT), inter-muscular adipose tissue (IMAT), and intra-pelvic gas (IPG). An authoritative radiologist finally checked the labeled data to minimize the individual errors.

In the preprocessing phase, the image edges were randomly cropped according to pelvic region of ground-truth, randomly splitting the Multi-tissue Pelvic dataset into 80 images for training, 15 images for testing, and 5 images for validation.

#### 3.5.2. CTPelvic1k Dataset

The CTPelvic1k dataset comes from a public pelvic 3D CT dataset. Selecting the first 70 pelvic fracture CT from subset-6 with mean size of 512 × 512 × 345 voxels to form the dataset. The pelvic regions were annotated as five categories in the ground-truth: background, sacrum, left hip (LH), right hip (RH), and lumbar spine (LS). A random 50 CT were assigned to the training set, 10 to the validation set, and 10 to the test set. Finally, a centering crop was used to reduce the size of the 3D CT volume.

#### 3.5.3. ACDC Dataset

The ACDC dataset is hosted in MICCAI 2017 open challenge and contains of 200 3D short-axis MRI scans from 100 patients, along with the expert annotations for three cardiac regions: myocardium (Myo), left ventricle (LV), and right ventricle (RV). Following existing work [39], this experiment used a split with a ratio of 140:20:40 for training, validation, and testing. All the 3D voxels were resized into 256 × 256 pixels and readjusted for intensity per slice to *[0,1]*.

### 3.6. Implementation Details

#### 3.6.1. Network Architectures

For all experiments on the same dataset, the U-Net [40] model was fixed as the baseline for comparison. For the training experiments on Multi-task Pelvic, ACDC dataset, and CTPelvic1k dataset, this work considered 2D, 2D, and 3D U-Net models, respectively. The U-Net structure consisted of a convolution layer, followed by a normalization layer InstanceNorm, and finally, an active layer ReLu.

#### 3.6.2. Training Procedure

All the experiments were implemented with PyTorch under an NVIDIA GeForce RTX 3090 and trained by the Stochastic Gradient Descent (SGD) optimizer with a momentum of 0.9 and weight decay of 10^−4^ and a poly-learning rate strategy with an initial learning rate of 0.01. The hyper-parameter α of interpolation factor was set to 0.2 [16]. For semi-supervised learning, this work set up a two-stream batch input strategy. The batch sizes of ACDC, CTPelvic1k, and Multi-task Pelvic dataset were set to 8, 4, and 4, respectively.

To alleviate the over-fitting of networks, we employed the random flip, rotation data, and random clipping augmentation techniques [41,42]. The ACDC, CTPelvic1k, and Multi-tissue Pelvic dataset were fed into a network with patch sizes of 256 × 256, 112 × 112 × 112, and 256 × 256, respectively. In the inference phase, a sliding window was used to obtain the final result of CTPelvic1k and Multi-tissue Pelvic with a corresponding stride of 64 × 64 × 64 and 64 × 64. For ACDC, predictions were generated slice-by-slice in the form of 2D and were stacked into a 3D-volume form.

### 3.7. Evaluation Criteria

To quantitatively evaluate the segmentation performance, the experiments followed the two commonly used complementary evaluation metrics for the ACDC dataset: DSC and Hausdorff Distance (HD). DSC is defined as the degree of overlap between the segmentation region and the ground-truth region, which is an index to measure the region mismatch and is expressed as

(8) $$DSC\left(G,S\right)=\frac{2\left|S\cap G\right|}{\left|S\right|+\left|G\right|},$$

where $DSC\in \left[0,1\right]$; the higher the value of DSC, the better the segmentation performance. S is the predicted labels, G is the corresponding ground-truth labels, $\left|S\cap G\right|$ is the intersection of S and G.

HD is a boundary-based metrics, which is used to measure the boundary errors, and can be defined as follow:

(9) $$HD\left(G,S\right)=max\left\{d_{SG},d_{GS}\right\}=max\left\{\underset{s\in S}{\max}\;\underset{g\in G}{\min}\;d\left(S,G\right),\underset{g\in G}{\max}\;\underset{s\in S}{\min}\;d\left(G,S\right)\right\},$$

where $d_{SG}$ is the largest distance of nearest-neighbor from points in S to G. Here, taking 95% of HD to eliminate the influence of a small subset of outliers and denoted as $HD_{95}$ [16,21,38].

For the CTPelvic1k dataset, experiment performances are evaluated by using the region based metrics DSC as mentioned above.

For the Multi-tissue Pelvic dataset experiments, the three metrics used include DSC, mean Intersection over Union (mIoU), and mean Accuracy (mAcc). The mIoU is defined as

(10) $$mIoU\left(G,S\right)=\frac{\left|S\cap G\right|}{\left|S\cup G\right|},$$

where $\left|S\cup G\right|$ is the union of S and G.

mAcc is a pixel-level metric, which indicates the percentage of pixels with correct prediction in all pixels:

(11) $$mAcc=\frac{TP+TN}{FN+TP+FP+TN},$$

where TP is true positive, TN is true negative, FN is false negative, and FP is false positive.

## 4. Results

### 4.1. Ablation Analysis of ACDC

#### 4.1.1. Ablation Experiments of Related Works

Under the experimental setup with a batch size of eight and three labeled samples, two evaluation metrics are shown in Table 2. The first section lists the comparison of related works, and the second section gives the choice of pseudo-label supervision loss.

**Figure 4:**
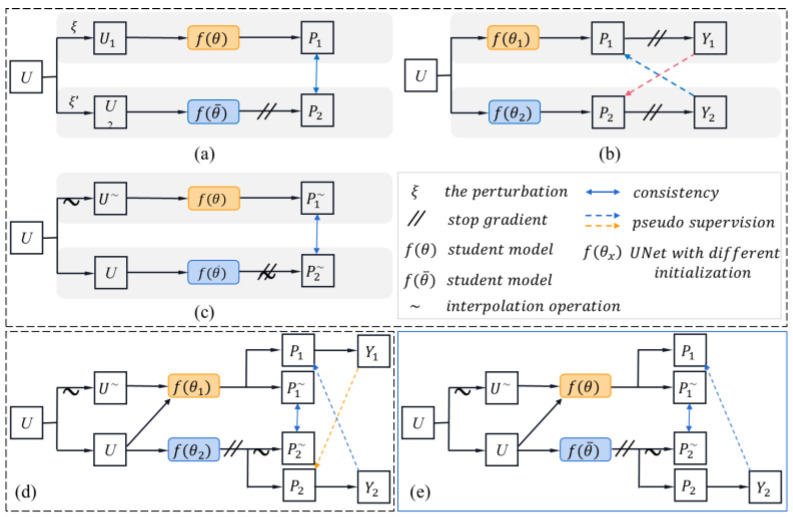
Illustration of the diagrammatic architecture for related works; only the semi-supervised part is given here. (**a**) mean teacher, (**b**) cross-pseudo-supervision, (**c**) interpolation consistency training, (**d**) cross-pseudo-interpolation consistency learning, (**e**) pseudo-interpolation consistency training. (**a**–**c**) are the related advanced works, (**d**,**e**) are the proposed schemes. U denotes the unlabeled data, P is the prediction map, Y represents the pseudo-label, $f\left(\theta_{1}\right)$ and $f\left(\theta_{2}\right)$ represent network with different initialization. MT can be considered as the baseline for all work. Here, MT’ is used to represent two networks with different initialization. Therein, (**b**) can represented by MT’ + CP, (**c**) can be denoted as I + MT, (**d**) can be denoted as MT’ + CP + I, and (**e**) can be denoted as I + MT + P.

From the experiment results, the PICT with the best DSC score outperforms all the related works. The diagram illustration of the related work is shown in Figure 4. MT can be seen as the basic framework for other semi-supervised methods. The CPICT is based on CPS and adds an auxiliary interpolation task. Experimental results showed that the DSC was significantly improved by around 4%. However, CPICT needed to train two networks with different initialization, which is time-consuming. Considering the high time requirement for clinical applications, the PICT is based on simple and efficient ICT and adds a pseudo-supervision module. The time cost caused by the auxiliary task of pseudo-supervision can even be conditionally ignored in small batch size. PICT further improves segmentation performance at a low time cost. The experimental results of CPICT and PICT show that the joint interpolation consistency module and the pseudo-supervision module are practical. These two modules focus on the original feature structure of unlabeled data and its interpolation augmentation data, which can complement each other to improve the ability of capturing detailed features.

The second section discusses the influence of pseudo-loss function. The experiment results show that when using the same CE loss as CPS, the optimization effect is not apparent, as shown in PICT (CE). When the PICT (CE + Dice) uses such a supervised loss manner in the combination of CE loss and Dice loss as the pseudo-supervision loss, the performance of DSC further improves by around 2%. Thus, it is reasonable to speculate that Dice may be more effective as a medical-image pseudo-supervision loss. Then, we tried only using Dice loss as a pseudo-loss, namely PICT (Dice); as the result was greatly improved, we finalized it as the final plan model.

**Figure 5:**
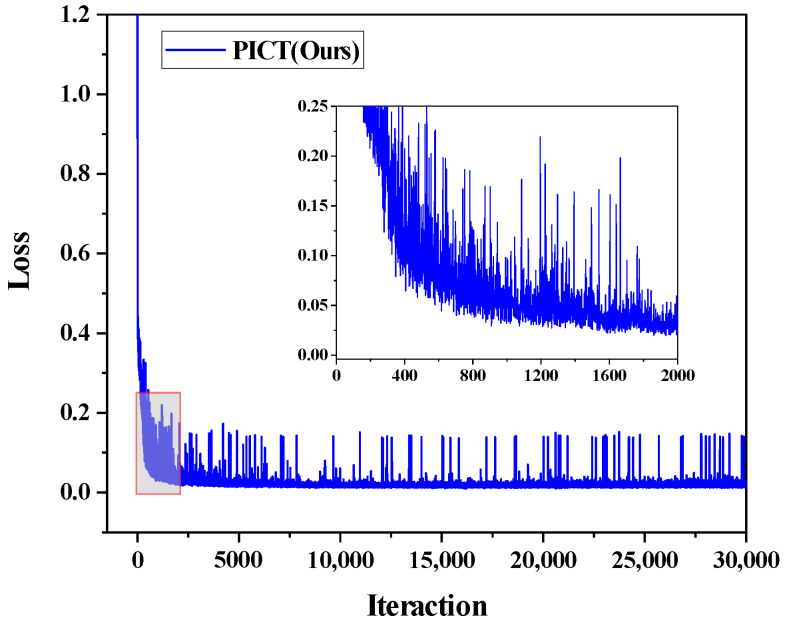
The loss function of the proposed PICT on ACDC dataset with three labeled cases.

Figure 5 shows the loss function during the three labeled case-training processes. It shows that the loss function of the proposed model converges well.

#### 4.1.2. Ablation Experiments of Backbone Models

Taking the examples of seven labeled cases, three common models (E-Net [43], P-Net [44], and U-Net [40]) were compared to discuss their generalization ability. As listed in Table 3, the fully supervised training can be regarded as the lower limit and denoted as LS. Compared with the fully supervised backbone model, the proposed semi-supervised framework improves by 3–9% by the utilization of additional free unlabeled data, showing its potential for the utilization of unlabeled data. Compared with ICT and CPS, PICT achieves the best DSC score on the three models, showing its generalization ability in adapting different models. For a fair comparison, the subsequent experiments will use the same U-Net backbone model as [16,21,39,45] to verify the effectiveness of the proposed semi-supervised module with unlabeled data.

#### 4.1.3. Comparison with Baseline and Existing Methods

We compared the proposed method PICT against baselines, the latest six methods, and the fully supervised method. The baselines and fully supervised refer to the low-bound performance and upper-bound performance of the used 3D U-Net model on labeled data and are denoted as LS and FS, respectively. The latest methods include Interpolation Consistency Training (ICT) [16], Mean Teacher (MT) [33], Cross-Pseudo Supervised (CPS) [35], Uncertainty Aware Mean Teacher (UAMT) [21], Cross Teaching between CNN and Transformer (CNN-Trans) [39], and Uncertainty Rectified Pyramid Consistency (URPC) [45]. For the semi-supervised method, MT method can be regarded as a prototype and semi-supervised baseline.

Table 4 lists the results comparison of using three labeled cases (six volumes) and seven labeled cases (14 volumes). Report values show that all the semi-supervised methods yield improvements over the supervised baseline LS and semi-supervised baseline MT. For three labeled cases, PICT largely improves the DSC score from 61% of LS and 67% of MT to 81.9% compared with the two baselines, and improves it by 10–15% in comparison to the latest methods. For seven labeled cases, the ICT and CPS improved by 0.6% and 0.3% in comparison with MT. The improvement confirms that these two kinds of models are effective. The proposed improved approach PICT based on ICT and CPS further outperforms MT by 1.2% and achieved the most advanced performance of both mean DSC score and ${\mathrm{HD}}_{95}$ with 87.18% and 5.46 mm, respectively. The minimum ${\mathrm{HD}}_{95}$ indicates that our method has the ability to regularize dividing boundaries and avoid large gaps in the real area of the ground-truth.

**Figure 6:**
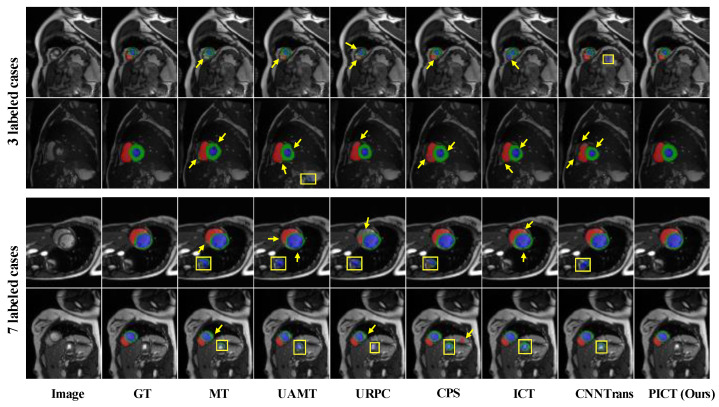
Visual comparison on ACDC test images of the state-of-the-art methods. The top two rows are the results of using 3 labeled cases; the bottom two rows are the results of using 7 labeled cases. The yellow arrows indicate the misclassify situation of target; the yellow boxes indicate the misprediction in other area.

As shown in Figure 6, the visualization results can more intuitively reflect the model performance. It can be observed that the segmentation prediction of our method is closer to the ground-truth mask compared with other methods. These experiment results on ACDC show the potential of the proposed semi-supervised method in alleviating the label cost.

### 4.2. Performance Comparison on CTPelvic1k

**Figure 7:**
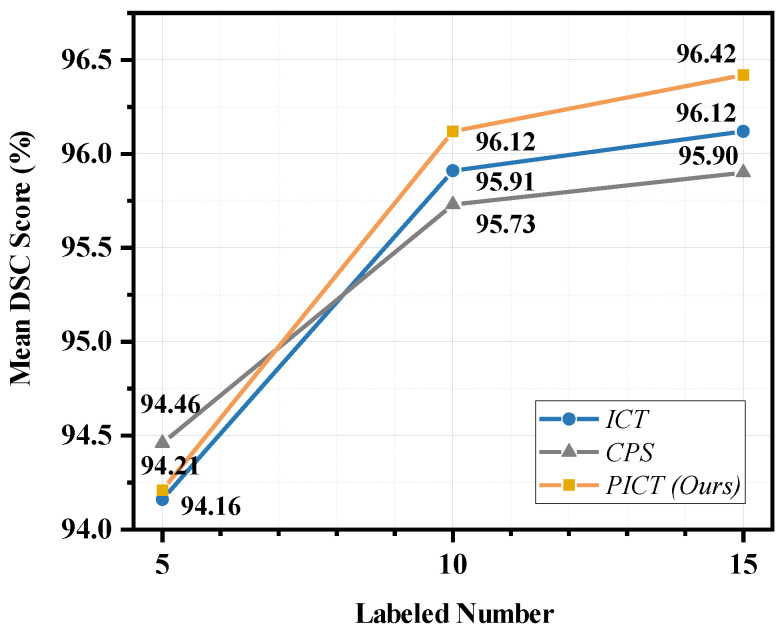
The mean DSC score point-fold line of different methods on CTPelvic1k dataset with different ratio labeled cases.

The blurry edges caused by fracture are the major challenge for the accurate segmentation of pelvic CT. In order to investigate the performance of the proposed technique, we set three comparisons with different labeled levels on the fracture dataset CTPelvic1k, as seen in Figure 7. From the comparison results, our method still has certain advantages in most cases. The mean DSC scores of CPS, ICT, and PICT are 95.73%, 95.91%, and 96.12%, respectively. As the labeled cases were up to 15, the corresponding mean DSC score further improved to 95.90%, 96.12%, and 96.42%, respectively. Taking 10 labeled data points as an example, the DSC score comparison of four categories are listed in Table 5. Results show that PICT achieved the highest performance in all categories except sacrum. This work did not use any preprocessing as in other pelvic segmentation studies, such as oversampling operations, to deal with the severe imbalance between the sacroiliac joint and lumbosacral joint [36]. All the contributions come from the model itself.

**Figure 8:**
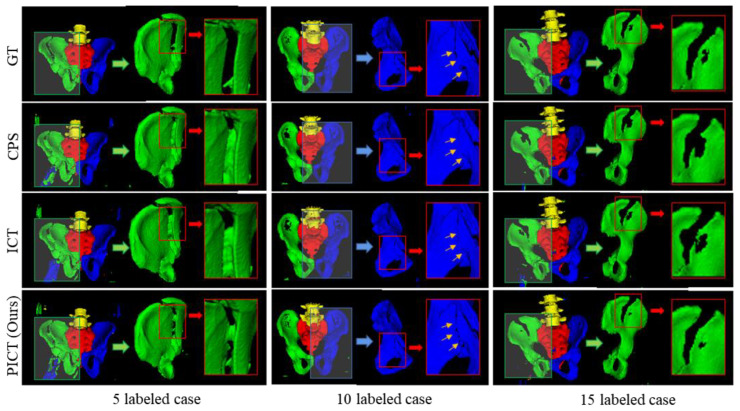
Representative cases of three methods on CTPelvic1k dataset. The green, blue, yellow, and red parts of the visual model represent the left hip bone, right hip bone, lumbar spine, and sacrum, respectively.

The 3D visualization of the segmentation results is convenient for doctors to know the fracture types at a glance. Figure 8 visually shows several typical comparisons of each method. It can be observed from the figure that all the models performed well on fracture pelvic CT. When paying attention to the 3D pelvic model itself, there was little difference from the ground truth. However, in small, labeled cases like 5, these three methods have different degrees of noise segmentation phenomenon, and the noise segmentation in ICT is the most obvious, followed by PICT. The reason may be that both ICT and PICT take disturbance data as input, which may bring some unpredictable noise. As the number of labeled images increases, PICT shows a higher DSC score and less noise segmentation. This may be due to the advantages of yhe pseudo-supervision part, and the chosen loss function is more suitable for medical images than CPS. The experiment shows that the proposed PICT holds for 3D pelvic fracture CT analysis.

### 4.3. Performance Comparison on Multi-Tissue Pelvic1k

**Figure 9:**
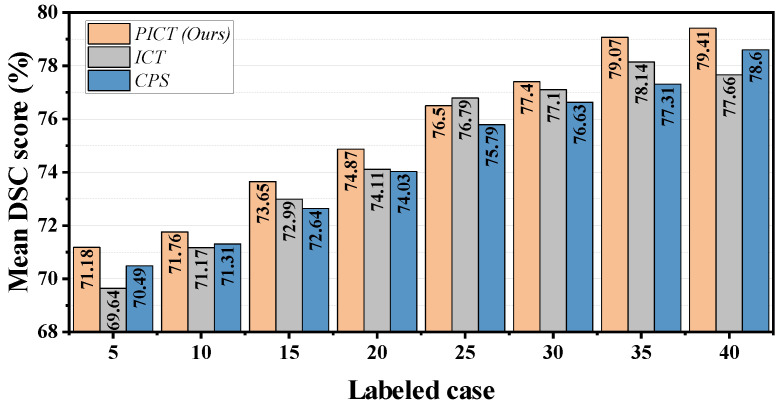
The mean DSC score point-fold line of different methods on Multi-tissue Pelvic dataset with different ratio labeled cases.

This paper further studied the segmentation performance of the proposed method on Multi-tissue Pelvic. To demonstrate superiority, this section quantitatively studied the performance under different labeled ratios; the mean DSC score comparison of the six categories is shown in Figure 9. From the histogram, the proposed method improves consistently over almost every labeled–unlabeled ratio. Compared with ICT and CPS, the combined model PICT has achieved about 1% gain on average in these eight labeled cases. The comparative analysis of the other two indicators mIoU and mAcc are listed in Table 6. Similarly, PICT has an advantage of around 1% over the baseline model in most cases. In the case of 40 labeled images of PICT, these three metrics (DSC, mIoU, and mAcc) reached 79.4%, 71.1%, and 81.8%, respectively. We compare this work with [45], which also studied pelvic tissue and muscle segmentation. They discuss a fully supervised model with 540 CT slices as input and reported that the corresponding three metrics were 74.9%, 63.6%, and 76.6%, respectively. The model proposed in this paper shows excellent advantages in each indicator and the amount of training data. This work is expected to further introduce a three-dimensional visualization model for simultaneous segmentation of multiple tissues of the pelvis.

**Figure 10:**
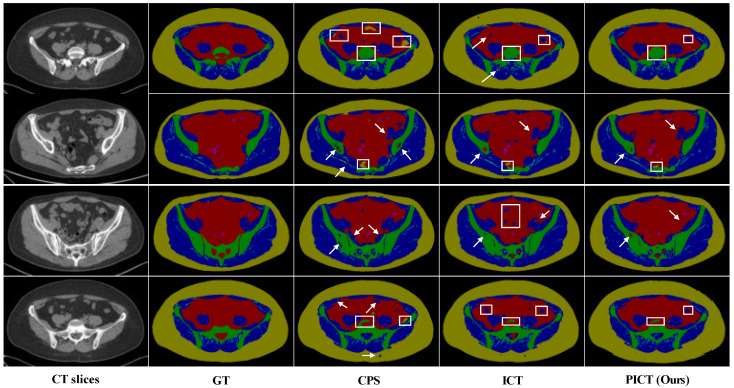
Visual comparison of the state-of-the-art methods on Multi-tissues Pelvic test images. The top two rows are the results of using 35 labeled cases; the bottom two rows are the results of using 40 labeled cases. The white arrows and boxes indicate the misclassified pixels.

To further investigate the feasibility of the proposed method, the comparison of each category’s performance with 40 labeled as an example are listed in Table 7. The proposed PICT achieved the advantages of three metrics in most cases. Figure 10 visuallys shows the comparison. We randomly selected several test images as examples; the visualization results show that the predictions of the proposed PICT are closer to the ground-truth in comparison with ICT and CPS. Similarly, this experiment did not use any post-processing, including dilation and fixed thresholding. Therefore, there will be some mispredictions inside the bone.

The proposed algorithm improves consistently over almost every category under every labeled–unlabeled ratio. The segmentation performance results show the ability of PICT in 2D pelvic CT analysis with multi tissues.

### 4.4. Training Time Costs

Finally, we list the time spent on three datasets during training processes in Table 8. Table shows that the lowest time cost was for ICT, followed by PICT, and finally CPS. The proposed PICT achieved state-of-art performance with a more acceptable time cost than CPS.

## 5. Discussion

Based on the above quantitative and qualitative results, it can be found that PICT achieves state-of-the-art performance and efficiently reduces the costs of medical image research. The ablation experiments on the open ACDC dataset showed that the combination of the interpolation module and pseudo-supervision module is effective. The pseudo-supervision module can constrain some ineffective interpolation perturbations where pixels are prone to misclassify. The data augmentation of pixel interpolation can make up for the unstable quality of the pseudo-label. This idea is also confirmed on two pelvic datasets: CTPelvic1k and Multi-tissue dataset.

Facing medical image segmentation with different types and tasks, the PICT proposed in this paper still has certain advantages. These results show that the proposed method is progressive and generalizable. This has clinical significance for exploring pelvic data analysis and other medical data analysis.

However, the proposed PICT also has a limitation. Manually annotating a multi-tissue 3D pelvic CT dataset is associated with difficulties, even when training in a semi-supervised manner. In future work, we plan to develop a 3D pelvic dataset containing multiple tissue and organs. For the semi-supervised model, we plan to automatically balance the weights of the interpolation consistency part and pseudo-supervision part.

## 6. Conclusions

This paper proposed a bi-direction constrained dual-task consistency semi-supervision method named PICT for few-label medical images, consisting of an interpolation consistency regularization task and a pseudo-supervision task. It can leverage free unlabeled data to capture more tissue semantic feature in the low contrast area and is sensitive to fracture margins with low time cost. The experiments on ACDC 2018, CTPelvic1k, and Multi-tissue datasets proved that the proposed PICT achieves state-of-the-art performance in comparison to the latest semi-supervised methods. This model can be used to visualize the anatomical morphology of soft hard tissue in computer-assisted surgery and can promote some automatic operations such as automatic path planning and postoperative evaluation, which is of great significance to promoting the application of machine learning in the clinical treatment of pelvic fractures.

## Figures and Tables

**Table 1:** Dataset statistics of CTPelvic1k, Multi-tissue Pelvic, and ACDC.

Parameters	Multi-Tissue Pelvic	CTPelvic1k	ACDC
Mean size	512 × 512	512 × 512 × 345	256 × 256 × 10
Data number	100	70	200
Tra/Val/Ts	80/5/15	50/10/10	140/20/40
Category	7	5	4
Type	Individual	Public	Public
Pre-process	Center crop	Center crop	None
Patch size	256 × 256	112 × 112 × 112	256 × 256

**Table 2:** Ablation analysis results on ACDC dataset using 3 labeled cases. The bold fonts indicate the best results of the comparing variants.

Method	RV		Myo		LV		Mean	
	DSC $\left(\%\right)$	$HD_{95}$ $\left(\%\right)$	DSC $\left(\%\right)$	$HD_{95}$ $\left(\%\right)$	DSC $\left(\%\right)$	$HD_{95}$ $\left(\%\right)$	DSC $\left(\%\right)$	$HD_{95}$ $\left(\%\right)$
MT [33]	56.77	37.83	67.76	16.61	76.46	24.37	67.00	26.27
CPS (MT’ + CP) [35]	61.46	18.59	70.90	12.54	78.67	**18.73**	70.34	**16.62**
ICT (I + MT) [16]	57.49	20.28	71.17	14.93	79.69	25.09	69.45	20.10
CPICT (CP + I + MT)	58.22	**16.91**	74.44	11.00	**81.34**	24.67	71.33	17.53
PICT (P + I + MT)	**63.27**	21.40	**77.90**	**10.63**	77.09	34.97	**72.76**	22.33
PICT (CE)	63.27	21.40	77.90	10.63	77.09	34.97	72.76	22.33
PICT (CE + Dice)	69.08	21.99	76.77	21.54	76.44	40.59	74.10	28.04
PICT (Dice, Ours)	**80.06**	**15.25**	**82.42**	**7.07**	**83.39**	**25.96**	**81.95**	**16.09**

**Table 3:** The DSC score (%) comparison of CPS, ICT, and PICT on ACDC dataset under different basic models with using 7 labeled cases. The bold fonts indicate the best results of the comparing variants.

Type of Basic Models	LS	CPS	ICT	PICT (Ours)
E-Net [43]	67.71	75.54	74.83	**76.66**
P-Net [44]	80.71	83.47	82.83	**83.76**
U-Net [40]	81.92	86.35	86.64	**87.18**

**Table 4:** Quantitative comparison results of mean DSC score and ${\mathrm{HD}}_{95}$ of the state-of-art method on ACDC under 3 labeled cases and 7 labeled cases. The bold fonts indicate the best results of the comparing variants.

Labled Number	Method	RV		Myo		LV		Mean	
		DSC $\left(\%\right)$	$HD_{95}$ $\left(\%\right)$	DSC $\left(\%\right)$	$HD_{95}$ $\left(\%\right)$	DSC $\left(\%\right)$	$HD_{95}$ $\left(\%\right)$	DSC $\left(\%\right)$	$HD_{95}$ $\left(\%\right)$
3 cases	LS (baseline)	48.11	45.76	62.76	23.27	72.06	24.87	60.98	31.30
	FS	91.15	1.23	88.62	5.95	93.58	5.62	91.12	4.26
	MT [33]	56.77	37.83	67.78	16.61	76.46	24.37	67.00	26.27
	UAMT [21]	57.86	32.10	67.32	14.57	76.00	20.81	67.06	22.49
	URPC [45]	63.73	33.13	69.59	15.95	79.19	18.61	70.89	22.56
	CPS [35]	61.46	18.59	70.90	12.54	78.67	18.73	70.34	16.62
	ICT [16]	57.48	20.28	71.17	14.83	79.69	25.09	69.45	20.06
	CNN-Trans [39]	57.70	21.70	62.80	11.50	76.30	**15.70**	65.60	16.20
	PICT (Ours)	**80.06**	**15.25**	**82.41**	**7.06**	**83.39**	25.96	**81.95**	**16.09**
7 cases	LS (baseline)	79.42	8.39	79.61	3.06	86.75	18.97	81.93	13.39
	FS	91.15	1.23	88.62	5.95	93.58	5.62	91.12	4.26
	MT [33]	86.31	4.73	83.39	8.81	88.32	17.11	86.01	10.22
	UAMT [21]	84.96	4.98	83.46	9.16	89.20	14.89	85.87	9.68
	URPC [45]	85.77	4.65	83.79	7.44	89.08	**7.44**	86.21	6.85
	CPS [35]	86.09	3.64	84.31	9.66	88.63	13.16	86.35	8.82
	ICT [16]	86.49	4.48	84.12	9.27	89.33	11.38	86.64	8.37
	CNN-Trans [39]	84.80	7.80	84.40	6.90	90.10	11.20	86.40	8.6
	PICT (Ours)	**86.74**	**3.22**	**85.16**	**3.24**	**89.66**	9.93	**87.18**	**5.46**

**Table 5:** Comparison of DSC score (%) of CPS, ICT, and PICT on CTPelvic1k with 10 labeled images as examples. The bold fonts indicate the best results of the comparing variants.

Categories	Sacrum	LH	RH	LS
CPS [35]	95.59	95.75	96.19	95.39
ICT [16]	**95.98**	96.05	95.82	95.81
PICT (Ours)	95.92	**96.09**	**96.44**	**96.03**

**Table 6:** The mIoU (%) and mAcc for CPS, ICT, and PICT on Multi-tissue Pelvic with different ratio labeled cases. The bold fonts indicate the best results of the comparing variants.

Labeled Case		5	10	15	20	25	30	35	40
mIoU (%)	CPS [35]	61.25	**62.38**	63.43	64.49	66.86	67.67	68.36	70.18
	ICT [16]	59.36	60.80	63.51	64.62	**67.63**	68.32	69.29	69.05
	PICT (Ours)	**61.83**	61.79	**64.22**	**65.56**	67.46	**68.49**	**70.60**	**71.07**
mAcc (%)	CPS [35]	**73.85**	**74.73**	75.15	77.15	78.56	79.62	79.11	80.97
	ICT [16]	72.77	74.13	75.65	76.65	**79.33**	79.71	80.31	80.53
	PICT (Ours)	73.84	74.30	**76.60**	**77.92**	78.91	**79.97**	**80.60**	**81.75**

**Table 7:** Comparison of DSC score (%), mIoU (%), mAcc (%) indexes of the 6 categories with 40 labeled images as an example on Multi-tissue Pelvic. The bold fonts indicate the best results of the comparing variants.

Categories		MIPC	Bone	Muscle	SAT	IMAT	IPG
DSC (%)	CPS [35]	80.76	93.44	86.03	92.16	52.36	**67.44**
	ICT [16]	78.99	93.58	86.25	90.97	52.55	63.67
	PICT (Ours)	**83.22**	**93.84**	**86.92**	**92.71**	**53.88**	65.89
mIoU (%)	CPS [35]	69.21	87.93	75.78	85.83	36.09	**66.24**
	ICT [16]	67.22	88.18	76.12	84.18	36.53	62.11
	PICT (Ours)	**72.54**	**88.62**	**77.17**	**86.78**	**37.51**	64.46
mAcc (%)	CPS [35]	73.78	93.33	91.39	92.60	**51.87**	**64.15**
	ICT [16]	70.35	93.40	92.80	91.93	55.20	60.36
	PICT (Ours)	**77.29**	**94.25**	**91.63**	**93.12**	50.89	64.06

**Table 8:** The time cost of ACDC. CTPelvic1k, Multi-tissue dataset; the unit is minutes.

Dataset	CPS	ICT	PICT (Ours)
ACDC	132	100	105
CTPelvic1k	753	500	586
Multi-tissue Pelvic	31	24	26

## Data Availability

The CTPelvic1k dataset [36] and ACDC dataset [37] are publicly available. The individual Multi-tissues Pelvic dataset in this study is available upon request from the corresponding author.
